# Comprehensive analysis of prognostic genes in gastric cancer

**DOI:** 10.18632/aging.203638

**Published:** 2021-10-22

**Authors:** Shaohua Huang, Liping Ma, Biyang Lan, Ning Liu, Wenwei Nong, Zhihu Huang

**Affiliations:** 1 Department of General Surgery, Affiliated Minzu Hospital of Guangxi Medical University, Nanning, China; 2 Department of Clinical Laboratory, Affiliated Minzu Hospital of Guangxi Medical University, Nanning, China

**Keywords:** gastric cancer, prognosis, apoptosis, biomarker, signaling pathways, immune cells

## Abstract

Background: Gastric cancer is associated with high mortality, and effective methods for predicting prognosis are lacking. We aimed to identify potential prognostic markers associated with the development of gastric cancer through bioinformatic analyses.

Methods: Gastric cancer-associated gene expression profiles were obtained from The Cancer Genome Atlas and Gene Expression Omnibus databases. The key genes involved in the development of gastric cancer were obtained by differential expression analysis, coexpression analysis, and short time-series expression miner (STEM) analysis. The potential prognostic value of differentially expressed genes was further evaluated using a Cox regression model and risk scores. Hierarchical clustering was applied to validate the impact of key genes on the overall survival of gastric cancer patients.

Results: A total of 1381 genes were consistently dysregulated in the development of gastric cancer. Among them, 186 genes affected the overall survival of gastric cancer patients. The following genes had areas under the receiver operating characteristic curve greater than 0.9 in both datasets and were therefore considered key genes: *ADAM12, CEP55, LRFN4, INHBA, ADH1B, DPT, FAM107A*, and *LOC100506388*. *LRFN4, DPT*, and *LOC100506388* were identified as potential prognostic genes for gastric cancer through a nomogram. Overexpression of *LRFN4* and *LOC100506388* was associated with a higher risk of gastric cancer. Finally, we found that tumors were infiltrated with high levels of Th2 cells and mast cells, and the infiltration levels were associated with overall survival in gastric cancer patients.

Conclusions: We found that key dysregulated genes may have a prognostic value for the development of gastric cancer.

## INTRODUCTION

Gastric cancer (GC) is the third leading cause of cancer-related deaths and the fifth most common type of malignancy worldwide [1]. About 50% of GC cases occur in East Asia, and it is the most common gastrointestinal malignancy in China [2]. Poor dietary habits, history of Helicobacter pylori infection, genetic factors, pre-malignant gastric lesions, and smoking have been identified as risk factors for gastric cancer [3]. Unfortunately, early gastric cancer diagnosis is not feasible in the majority of patients. As a result, most gastric cancers are diagnosed at advanced stages, and 25–50% of patients develop metastases during the course of the disease [4].

To date, the main treatment for gastric cancer includes surgical excision plus standard D2 lymph node dissection [5]. Currently, no chemotherapy or molecular therapy, alone or in combination with other treatments, consistently leads to an objective, lasting tumor response. Gastric cancer immunotherapy is another potentially effective therapeutic approach [6]. Although chemotherapy regimens have improved progression-free survival and overall survival in patients with advanced gastric cancer, their median survival tends to be less than one year [7]. The incidence of gastric cancer has decreased over the past decades, but the five-year survival rate remains only around 10% for patients with advanced disease [8]. Therefore, understanding the mechanism of disease progression and finding new and effective prognostic factors and targets for intervention are of great significance for improving long-term survival.

Gastric cancer is characterized by hypoxia and immunosuppression, thereby hindering the ability of the immune system to fight cancer [9]. Inflammatory mediators and cytokines play a crucial role in the formation of the tumor microenvironment, further stimulating tumor development [10].

In recent years, the application of high-throughput technologies has led to a new understanding of the molecular pathogenesis of GC, and potential markers and therapeutic targets can be explored based on the genomic characteristics of tumors [11]. In recent studies, many oncogenes or suppressor genes associated with GC have been reported [12, 13]. The protein “leucine rich repeat and fibronectin type III domain containing 4” (LRFN4) is expressed in a variety of cancers and leukemia cells [14]. LRFN4 signaling plays an important role in monocyte/macrophage migration [15]. Dermatopontin (DPT) can promote the formation of collagen fibers and improve the biological activity of TGF-β [16, 17]. The expression level of DPT may be closely related to the pathogenesis of cancer [18].

In the present study, we took gene expression data in GC from The Cancer Genome Atlas (TCGA) and Gene Omnibus (GEO) databases in order to search for prognostic genes and immune cells associated with GC development. To further explore dysregulated molecular mechanisms in GC, we performed enrichment analysis. Potential prognostic genes were evaluated using nomograms and risk scores. The effects of key genes on the survival of gastric cancer patients were validated by hierarchical clustering. These results may provide a new reference for predicting and understanding the prognosis of patients with gastric cancer.

## MATERIALS AND METHODS

### Data sources

We collected gastric cancer data from TCGA and GEO databases (GSE26942, GSE27342, and GSE66229). GSE26942 included gene expression profiling data from 205 gastric tumor tissues and 12 surrounding normal gastric tissues. Gene expression data were normalized by quantile normalization and log2 transformation. GSE27342 included gene expression profiling data of paired tumor and adjacent normal tissues from 80 gastric cancer patients. The raw probe intensities were normalized using the quartile normalization approach, and the probe signal was summarized to the level of gene expression using the pathway-level information extractor (PLIER) method [19]. GSE66229 included gene expression profiles from 300 gastric tumors and 100 normal controls. Raw expression data were normalized and summarized using the robust multichip analysis (RMA) method [20] along with log2 transformation. TCGA included RNAseq gene expression data of 373 cases of primary lung adenocarcinoma and 32 cases of normal samples. Count data were normalized using the DESeq2 package in R [21].

### Differentially expressed gene (DEG) analysis

DEGs between GC and controls in GEO datasets were identified using the limma package in R [22]. DEGs in the TCGA dataset were obtained using DESeq2. DEGs were defined as genes showing a > xx-fold difference with a *P*-value < 0.05.

### Co-expression analysis

Weighted gene coexpression network analysis (WGCNA) [23] was used to construct the gene coexpression network for DEGs. The soft-thresholding power we chose was used as the correlation coefficient threshold. The parameters were set as follows: minModuleSize = 30, verbose = 3, and mergeCutHeight = 0.25. Then, we built the minimum number of genes in gene modules. Module-trait correlation analysis was performed based on Pearson correlation.

### Enrichment analysis

Enrichment analysis of Gene Ontology (GO) terms and Kyoto Encyclopedia of Genes and Genomes (KEGG) pathways was performed for gene modules using the clusterProfile package in R [24]. GO terms included biological processes, cellular components, and molecular functions. ClusterProfiler was also used to conduct gene set enrichment analysis (GSEA) [25]. *P* value < 0.05 was chosen as the cut-off criterion for enrichment. Gene set variation analysis (GSVA) with KEGG pathways was performed using the GSVA Bioconductor package [26]. Activated or inhibited states of signaling pathways were calculated using limma.

### Single-sample GSEA

We obtained a set of marker genes for immune cell types from a previous publication [27]. The infiltration level of each immune cell type was calculated by single-sample GSEA using the GSVA tool in R. We calculated differences in immune cell infiltration between gastric cancer and control samples. Hierarchical cluster analysis was used to group tumors with different patterns of immune cell infiltration with the same invasion direction and significance for patients' survival.

### STEM analysis

First, we used the STEM algorithm and software (v1.3.11) to organize genes into distinct clusters based on expression patterns [28]. To filter out gene sets that were significantly correlated with the time series, the gene count in each cluster was set to >30, and the correlation coefficient of gene expression in each cluster was set to >0.8. All significant gene sets were associated with *P* < 0.05, and all showed similar expression trends.

### Identification of CpG sites and somatic mutation of GC

Infinium HumanMethylation450 BeadChip data of the GC and normal tissue samples were obtained from TCGA. The chAMP package [29] in R was used to identify differences in methylation between the GC and normal in TCGA. The β value of methylation was calculated for all CpG probes (sites) with a detection *P* value ≤ 0.05. DNA methylation level at each site was calculated based on the methylation signal intensity (M) and non-methylation signal intensity (U). Somatic mutations in GC samples in TCGA were identified using maftools [30].

### Prediction of gene prognostic value

Kaplan-Meier estimator and log-rank tests were performed using the functions surv, survfit, and survdiff in R. Multivariate Cox regression analyses were used to test the independent prognostic value of the genes using the survival package and coxph function in R. Areas under the receiver operating characteristic curve (AUCs) were calculated for those genes using the pROC package [31].

### Data availability

The datasets presented in this study can be found in TCGA and GEO databases (accession numbers GSE26942, GSE27342, and GSE66229).

## RESULTS

### Differential expression analysis of gastric cancer and normal tissue samples

**Figure 1:**
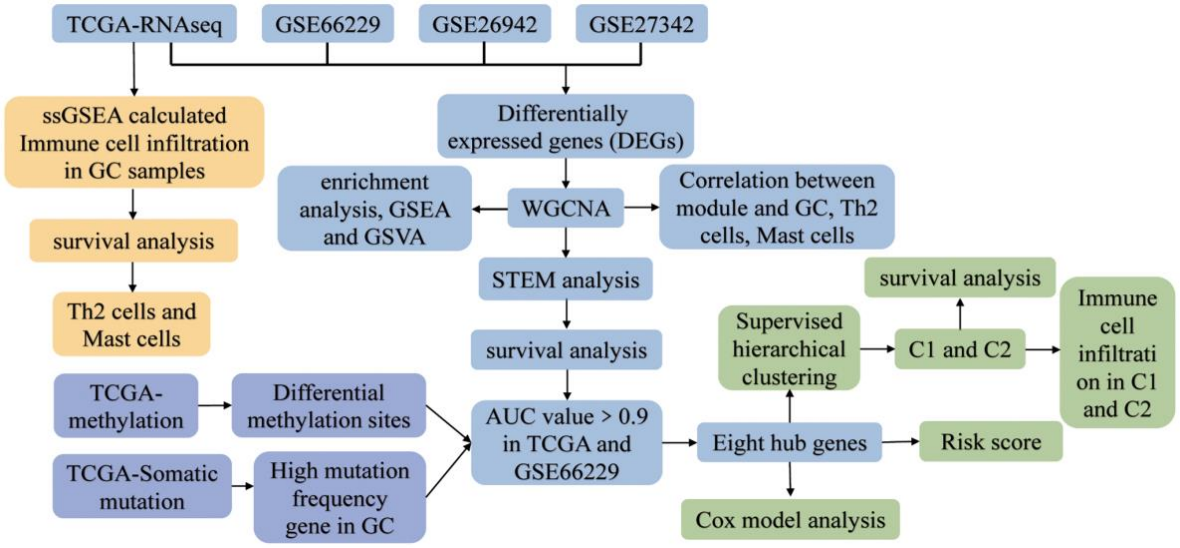
**The study flowchart.** Abbreviations: AUC: area under the receiver operating characteristic curve; C1: cluster 1; C2: cluster 2; GC: gastric cancer; GSEA: gene set enrichment analysis; GSVA: gene set variation analysis; ssGSEA: single-sample gene set enrichment analysis; STEM: short time-series expression miner; TCGA: The Cancer Genome Atlas; WGCNA: weighted gene co-expression network analysis.

**Figure 2:**
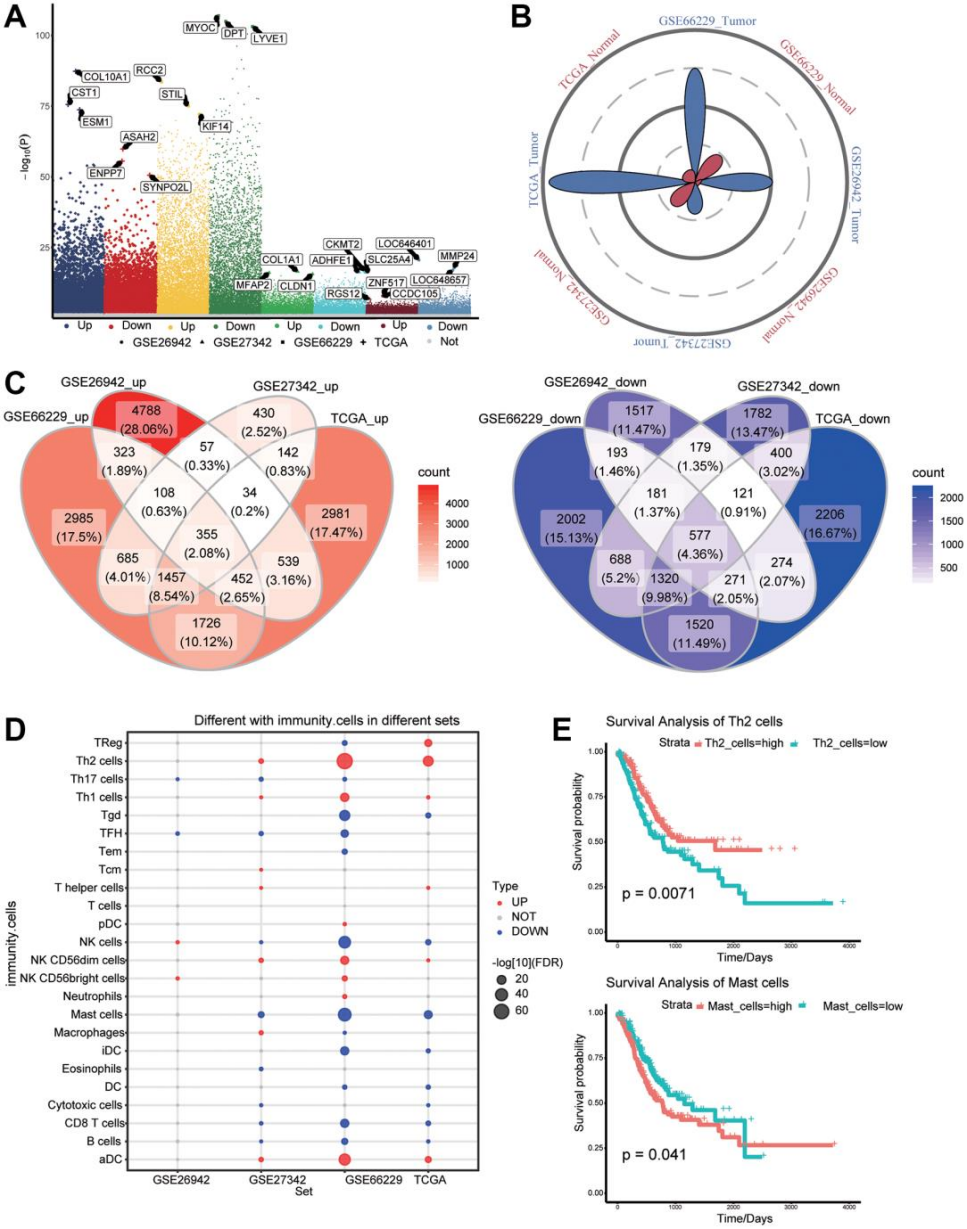
**Differentially expressed genes (DEGs) and immune cell infiltration in gastric cancer and normal tissues.** (**A**) DEGs between gastric cancer and normal tissues in The Cancer Genome Atlas (TCGA) as well as GSE26942, GSE27342, and GSE66229 datasets. (**B**) Petal plots of sample size for four sets of gastric cancer-related data. (**C**) Overexpressed (left panel) and under expressed (right panel) genes considered as DEGs in the TCGA data. (**D**) Differential infiltration of immune cells between gastric cancer and normal tissues. (**E**) Kaplan–Meier curves showed that immune cell infiltration was related to overall survival of gastric cancer patients.

The flowchart of the study is shown in Figure 1. According to the screening criteria of DEGs, we obtained 14375 DEGs in TCGA data, 9969 DEGs in GSE26942, 8516 DEGs in GSE27342, and 14843 DEGs in GSE66229, all with *P*-values <0.05 (Figure 2A). The sample sizes of TCGA and GSE66229 were relatively large (Figure 2B). In these two datasets, 9,188 common DEGs (either up- or downregulated) were identified (Figure 2C).

### Immune cell infiltration

To identify the potential role of immune cells in gastric cancer, we analyzed differences in immune cell infiltration between tumors and normal tissues (Figure 2D). Among the differentially infiltrated immune cells, Th2 and mast cells affected the overall survival of gastric cancer patients (Figure 2E).

### Construction of coexpression networks for DEGs

**Figure 3:**
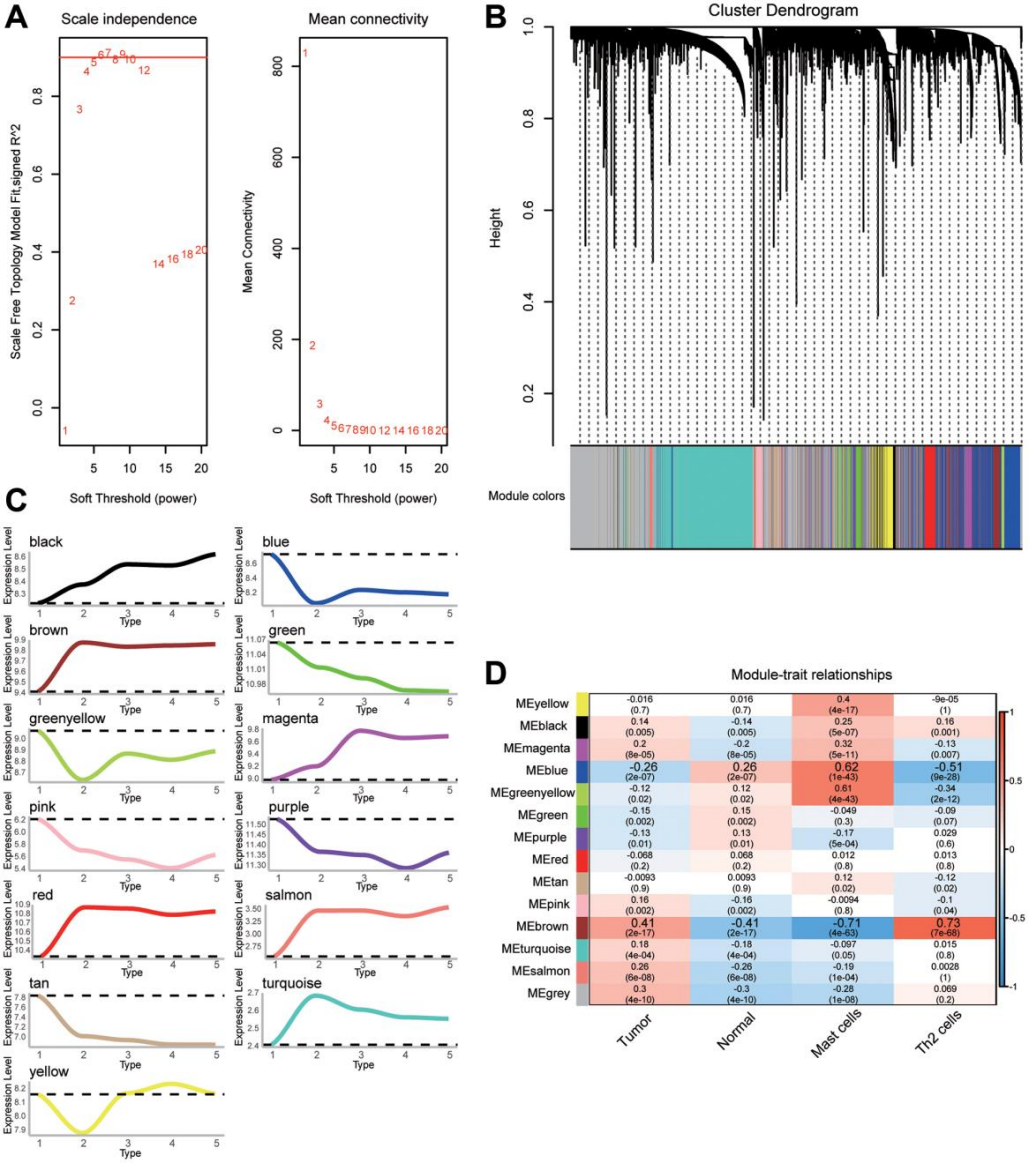
**The weighted gene coexpression network analysis (WGCNA) network for selected genes.** (**A**) Analysis of the scale-free fit index and the mean connectivity for various soft-thresholding powers (β). (**B**) Cluster dendrogram of differentially expressed genes (DEGs) showing similar expression trends. (**C**) Up- or downregulation trend of gene modules. (**D**) Heatmap of the correlation between gene modules and clinical traits.

Common genes with | log2(fold change) | >0.5 were further screened to construct the coexpression network. β = 6 was chosen as the soft threshold power to construct a scale-free network (Figure 3A). We identified a total of 13 modules containing 4440 genes (Figure 3B). By correlating the expression of genes in modules to different stages of gastric cancer, we identified up- or downregulated modules (Figure 3C). Furthermore, the relationship between the modules and the clinical traits was evaluated to identify hub modules (Figure 3D). Significant correlations were found between modules and gastric cancer, as well as immune cells. Notably, the module named as 'blue' had the highest negative correlation with tumors and Th2 cells, but the highest positive correlation with normal and mast cells. The correlation between the brown module and traits was the opposite.

### Biological functions of module genes

**Figure 4:**
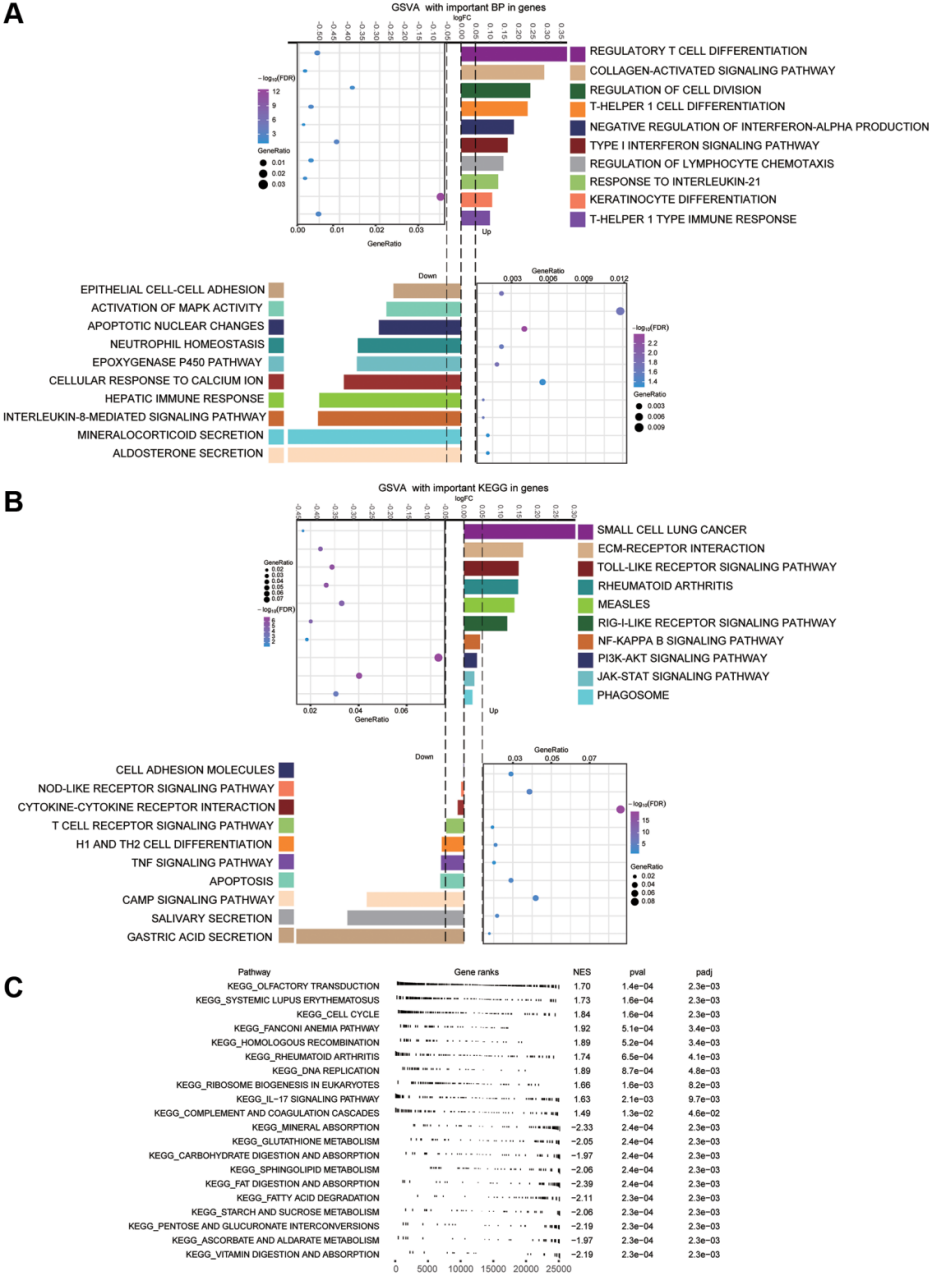
**Main enrichment results of module genes.** (**A**) Main biological processes (BPs) of module genes. (**B**) Kyoto Encyclopedia of Genes and Genomes (KEGG) pathway analysis for module genes. (**C**) Gene set enrichment analysis (GSEA) terms of up- and down-regulated genes. The first 10 terms corresponded to up-regulated genes, and the last 10 terms to down-regulated ones.

Performing enrichment analysis of module genes, we identified GO functions and KEGG signaling pathways associated with gastric cancer. Among the 1003 biological processes with *P*-value < 0.05, we found that “regulatory T cell differentiation”, “type I interferon signaling pathway”, “response to interleukin-21”, “inhibition of hepatic immune response”, “apoptotic nuclear changes”, and “activation of MAPK activity” were enriched (Figure 4A) Among the 116 KEGG pathways enriched for module genes, pathways involving PI3K-Akt, JAK-STAT, and Toll-like receptor (TLR) were activated, while pathways involving Th1 and Th2 cell differentiation, apoptosis, and tumor necrosis factor (TNF) were inhibited (Figure 4B). GSEA identified activation of the cell cycle and inhibition of gastric acid secretion in GC (Figure 4C).

### Persistently dysregulated genes in the development of gastric cancer

**Figure 5:**
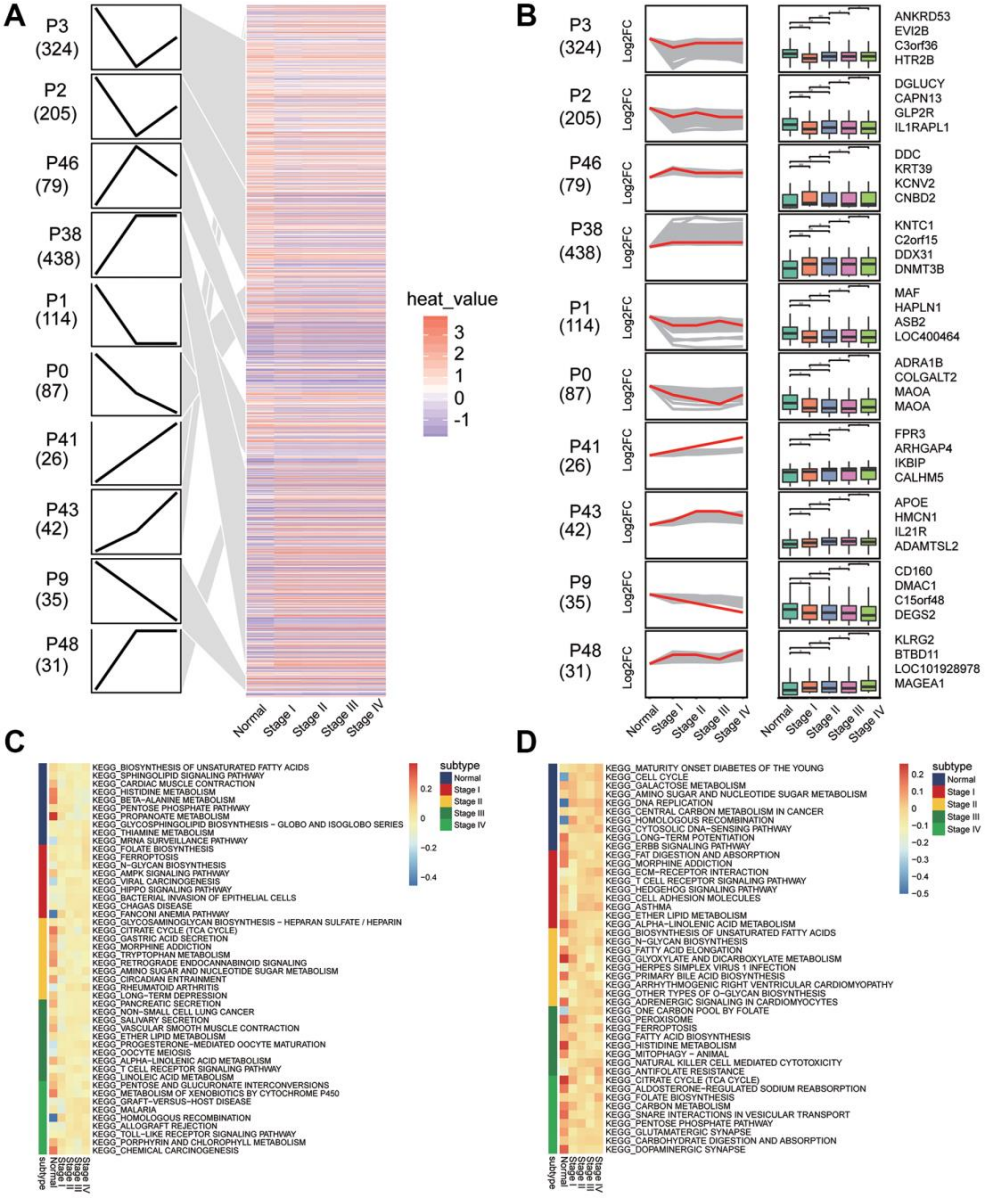
**Sets of genes showing persistent up- or down-regulation during gastric cancer development.** (**A**) Heatmap of gene sets with persistent under- or overexpression from normal to stage IV of gastric cancer. (**B**) The top four genes with the greatest fold-change in transcription level in 10 clusters. (**C**) Signaling pathways that are consistently up-regulated as tumors develop. (**D**) Signaling pathways that are consistently down-regulated as tumors develop.

Clustering of module genes by STEM software according to the development of gastric cancer revealed 10 groups of clusters with different dynamic gene expression patterns (Figure 5A and 5B). We found 1381 genes that exhibited consistently up- or downregulated expression patterns during the development of gastric cancer. SubtypeGSEA showed that TLR signaling pathway, mRNA surveillance pathway, and rheumatoid arthritis pathway were consistently activated with the development of gastric cancer, while histidine metabolism, alpha linolenic acid metabolism, and peroxisomes were consistently inhibited (Figure 5C and 5D).

### Key genes associated with gastric cancer progression

**Figure 6:**
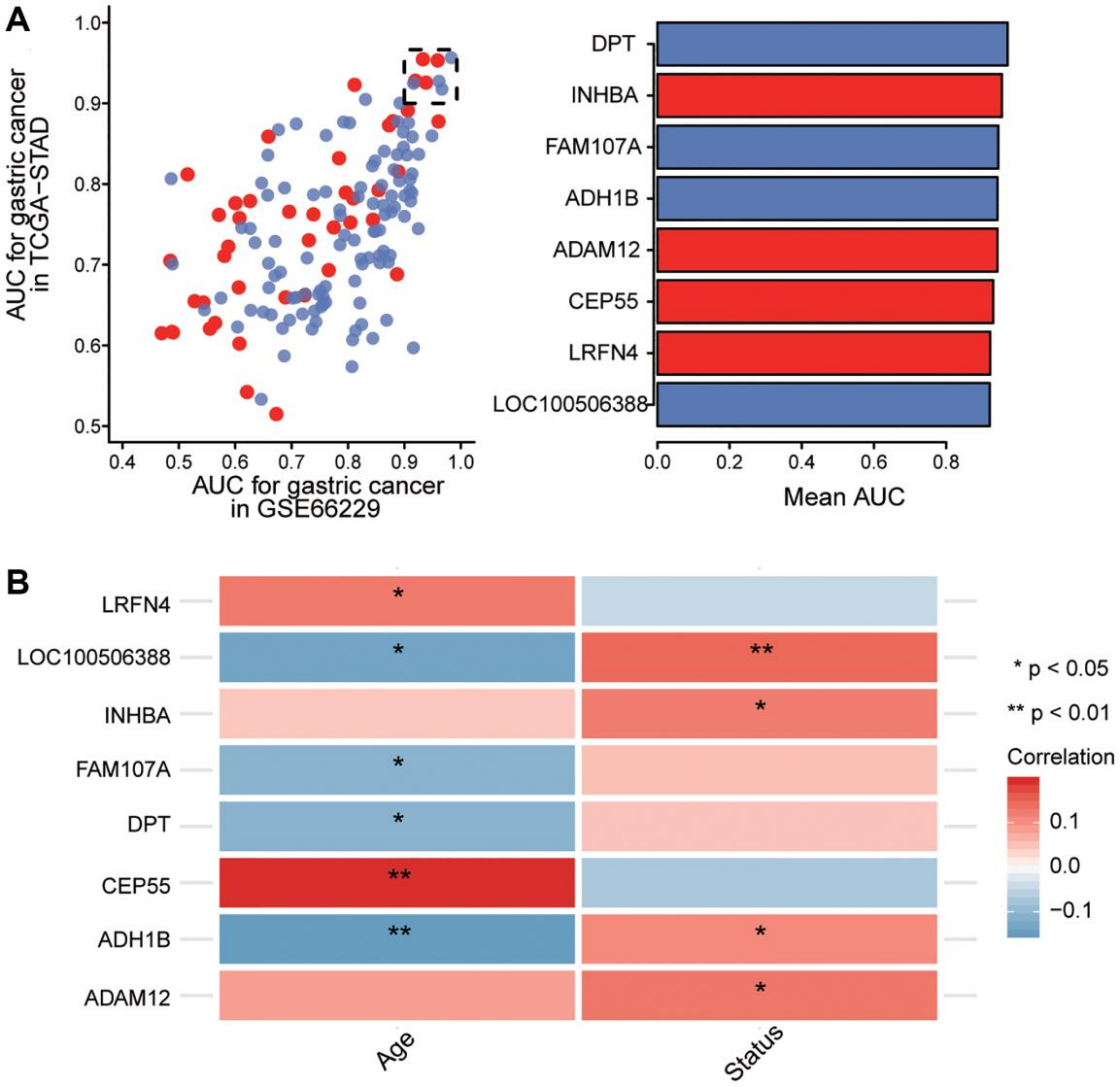
**Screening of key genes.** (**A**) Identification of key genes with areas under the receiving operating characteristic curve (AUCs) greater than 0.9 in The Cancer Genome Atlas (TCGA) and GSE66229. Red indicates up-regulated expression and blue, down-regulated. (**B**) Correlation of key gene expression with age and overall survival of gastric cancer patients in the TCGA dataset. Red indicates a positive correlation and blue, a negative correlation. ^*^P < 0.05, ^**^P < 0.01.

Principal component analysis found that the TCGA and GSE66229 data showed the greatest separation between tumor and normal samples (Supplementary Figure 1). We selected survival-related genes with AUCs greater than 0.9 as key genes (Figure 6A). Compared with normal tissues, *ADAM12, CEP55, LRFN4*, and *INHBA* were up-regulated in gastric cancer samples, while *ADH1B, DPT, FAM107A*, and *LOC100506388* were downregulated. In addition, our correlation analysis showed that key gene expression was significantly associated with age and overall survival of GC patients (Figure 6B). Interestingly, *ADH1B, DPT, FAM107A*, and *LOC100506388* belonged to the blue module, whereas CEP55 and LRFN4 belonged to the brown module.

### Prognostic model of key genes

**Figure 7:**
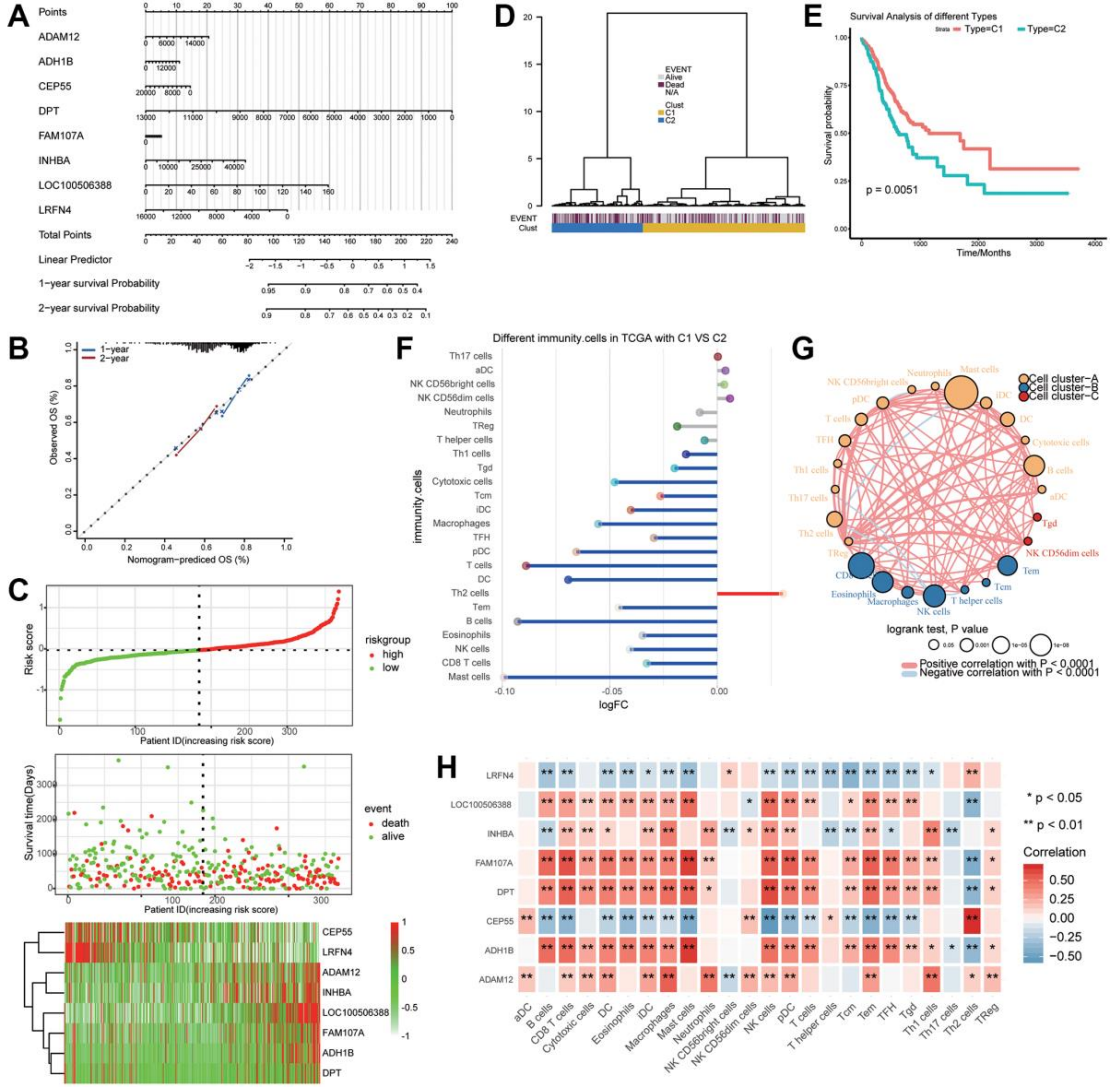
**Key genes associated with the prognosis of gastric cancer patients.** (**A**) The nomogram predicting overall survival in gastric cancer patients. (**B**) Plots depicting the calibration of the model in terms of agreement between predicted and observed one- and two-year outcomes. (**C**) Distribution of risk score, overall survival, and heatmap of the key genes in The Cancer Genome Atlas (TCGA) dataset. (**D**) Dendrogram of key genes in the TCGA dataset distinguished survival status by supervised hierarchical clustering. (**E**) Two clusters were associated with overall survival. (**F**) Differences in immune cell infiltration between clusters 1 (C1) and 2 (C2). (**G**) Correlation and clustering for immune cells. (**H**) Correlation between the expression of key genes and immune cell infiltration.

Survival information of gastric cancer patients was analyzed using a Cox regression survival nomogram (Figure 7A). The results showed that elevated expression of *DPT* and *LRFN4* predicted good prognosis, while overexpression of *LOC100506388* was associated with poor prognosis. The calibration curve showed that the nomogram performed well compared to an ideal model (Figure 7B).

Risk scores were calculated based on the expression of key genes, and the median risk score was used to classify patients into high- or low-risk groups. The results showed that *ADAM12, INHBA, LOC100506388, FAM107A*, and *ADH1B* were upregulated in the high-risk group, while *CEP55* and *LRFN4* were highly expressed in the low-risk group (Figure 7C).

Supervised hierarchical clustering of key genes that discriminated the prognosis of gastric cancer patients generated two clusters (Figure 7D) that were associated with overall survival (Figure 7E). Compared with cluster 2, cluster 1 gene expression in gastric cancer patients correlated with good prognosis. Interestingly, Th2 cells were upregulated in cluster 1, whereas mast cells were downregulated (Figure 7F). These immune cells clustered into three categories, with a significant negative correlation between Th2 cells and mast cells (Figure 7G). CEP55 expression was most positively associated with Th2 cells and negatively associated with mast cells (Figure 7H). FAM107A and ADH1B were positively associated with mast cells but negatively associated with Th2 cells.

## DISCUSSION

The identification of early diagnostic and prognostic markers has important diagnostic and therapeutic implications for patients with gastric cancer [32]. In this study, we searched relevant genes that may have an impact on the overall survival of gastric cancer patients by identifying those persistently dysregulated during tumor development. Then, we identified potential marker genes for the diagnosis and prognosis of gastric cancer. Importantly, based on the expression of key genes, high- and low-risk patients groups with different overall survival were established.

Our results showed that Th2 and mast cells were significantly infiltrated in gastric cancer patients. Strong infiltration by Th2 cells may be associated with early carcinogenesis and promote the activation of a tumor microenvironment that favors angiogenesis and metastasis [33]. Studies have shown that Th2 cell responses are associated with the development and progression of human gastric cancer [34]. Our analysis showed that infiltration by mast cells was lower in GC than in controls in all three datasets. This contrasts with reports that mast cell infiltration increases in gastric cancer tissues [35, 36]. This difference may be explained by the small sample size of normal tissues in our data. Survival analysis previously showed that mast cell infiltration was associated with worse prognosis in gastric cancer [37].

We screened DEGs with similar expression patterns by co-expression network analysis, and found significant correlations between them and gastric cancer or immune cells. The enrichment results of these genes showed that they were associated with immune inflammation and cancer-related biological functions. Recent studies have shown that T cell immunity may play an important role in the progression and prognosis of gastric cancer [38], including stage based on the “tumor, node and metastasis” system, depth of invasion, lymph node metastasis rate, and tumor immunity [39]. The proinflammatory cytokine interferon-β can exert antitumor activity by inhibiting angiogenesis, tumor growth, and metastasis [40]. The serum concentration of IL-21 in patients with gastric cancer is significantly higher than that in controls, so the cytokine may play some role in the development and progression of gastric cancer [41]. Activation of mitogen activated protein kinase (MAPK) p38 amplifies the inflammatory process, which in turn increases gastric cancer cell migration and invasion [42]. Inhibition of the JAK/STAT3 pathway in gastric tumor tissues reduces the inflammatory response, inhibits the inflammatory cytokines IL-1L, IL-6, and IL-1β, and decreases tumor volume [43]. In gastric cancer cells, activated PI3K/Akt signaling leads to NF-κB activation, which ultimately promotes cell migration and invasion [44]. TNF, a proinflammatory cytokine that can suppress some tumors when present at high concentrations, has attracted some attention as an anticancer therapy [45].

Given that gastric cancer is diagnosed at a late stage and has a poor prognosis, it is important to identify the molecular mechanisms and markers that influence its development. TLRs are recognized to be involved in different periods of gastric cancer progression and are gradually upregulated. Activation of TLR signaling pathways induces inflammatory cytokines and signaling pathways that play important roles in diseases such as cancer [46]. TLR2 is involved in the pathogenesis of gastric cancer, and high levels of TLR4 are also associated with a higher risk of this tumor type [47]. However, further studies are needed to determine the exact role of each TLR in the developmental mechanism of gastric cancer.

In the present work, comprehensive survival analysis and Cox regression analysis found that upregulation of *DPT* and *LRFN4* correlated with good prognosis in gastric cancer patients. DPT is significantly downregulated in gastric cancer tissues [48], and it may contribute to oral cancer metastasis [49]. DPT is also involved in the inhibition of proliferation of keratinocytes, osteosarcoma cells, and papillary thyroid carcinoma cells in mice [50]. The expression of LRFN4 protein in cancer tissues is higher than in paracancerous tissues and benign gastric disease tissues [2]. Patients with high expression of LRFN4 had a higher survival rate than a low-expression group, and thus the potential protective role of LRFN4 in gastric cancer should be further explored [51]. Although we found no evidence that *LOC100506388* affects prognosis in gastric cancer, our results suggest that expression of *LOC100506388* may help to predict the disease.

We established an eight-gene hierarchical clustering based on signature genes associated with survival in gastric cancer. Survival curves showed that the overall survival of patients expressing cluster 1 was significantly longer than that of patients expressing cluster 2. This clustering analysis further demonstrates the important role of these feature genes when evaluating the survival of gastric cancer patients.

Our study presents several limitations. The data for this study come from public databases and our results lack experimental validation. Our results should be validated in large studies. Our results considered only the developmental stage of gastric cancer and did not take into account other clinical features such as metastatic recurrence. More in-depth analysis is needed in future studies.

## CONCLUSIONS

This bioinformatic study identified potential prognostic genes related to the development of gastric cancer that may be useful as potential markers and therapeutic targets. Our data support the potential of key genes, especially *LRFN4*, as diagnostic and prognostic biomarkers of gastric cancer.

## Supplementary Materials

Supplementary Figure
