# Supplementary material for: Comprehensive analysis of prognostic genes in gastric cancer
Source: Aging (Albany NY). 2021 Oct 22;13(20):23637–51. doi: 10.18632/aging.203638 (PMC8580339; doi:10.18632/aging.203638)
Supplement: Supplementary Figure [file aging-13-203638-s001.pdf]

SUPPLEMENTARY FIGURE

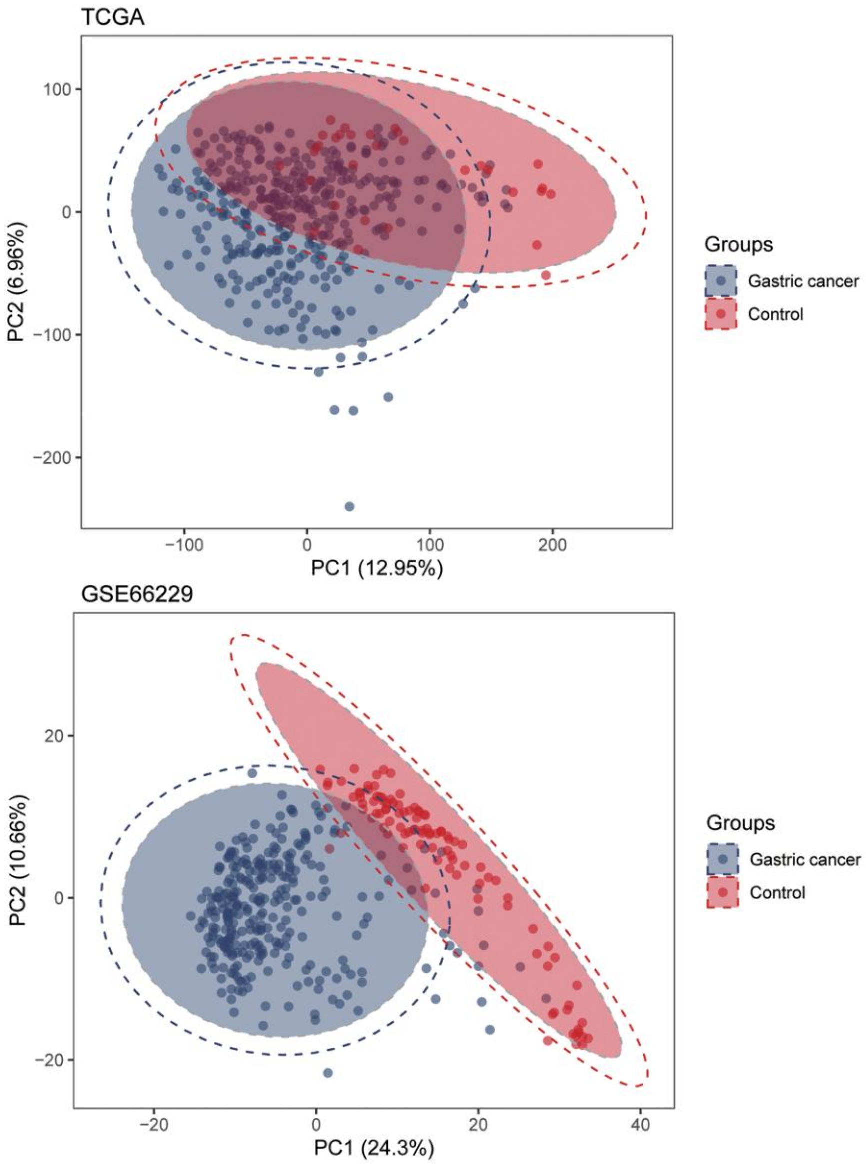

Supplementary Figure 1. The principal component analysis for gastric cancer samples in The Cancer Genome Atlas (TCGA) and GSE66229 datasets. PC1: Principal component 1; PC2: Principal component 2.
